# A CD4/Foxp3/OX40 triple immunofluorescence assay determines association between T cell immune subsets and outcome in colorectal cancer

**DOI:** 10.1186/2051-1426-2-S3-P133

**Published:** 2014-11-06

**Authors:** James Ziai, Mahrukh Huseni, Oded Foreman, Jeffrey Eastham-Anderson, Yuanyuan Xiao, Felix Chu, Marcin Kowanetz, Priti S Hegde, Jeong Kim

**Affiliations:** 1 Genentech, Inc., South San Francisco, CA, USA

## Purpose

Increased numbers of OX40+ T cells in the tumor microenvironment of colorectal cancer (CRC) patients have been associated with improved outcome. However, the OX40+ T cell population is heterogeneous and includes, among others, CD4+Foxp3+ regulatory T cells (Treg) as well as CD4+Foxp3- effector T cells (Teff). In this study, we used a novel triple immunofluorescence assay for CD4, FoxP3 and OX40 to determine the relationship between tumor-associated CD4+ T cell subsets and patient disease stage as well as outcome in CRC.

## Methods

We investigated OX40 expression in tumor-associated CD4+ T cell subsets in 48 CRC patients including primary site (n = 48) and matched metastases (n = 19) with a triple immunofluorescence assay for CD4/FOXP3/OX40. Stained sections were digitally imaged and single, double and triple-positive tumor-associated cell subsets were enumerated and associations between CD4 subsets and stage and outcome as well as that of CD4 subsets between primary site and metastases were determined.

## Results

Mean counts of total OX40+cells (p = 0.005), OX40+ Treg cells (p = 0.006), and OX40+ Teff (p = 0.019) cells showed statistically significant inverse correlation with increased stage at diagnosis. Associations remained statistically significant when counts were normalized to total cells. Total CD4+ or Foxp3+ cells did not show significant association with stage. Analysis of paired primary and metastatic samples (n = 19) showed strong correlation between primary and met positive counts for CD4 (r = 0.75), total OX40+ (r = 0.84) as well as OX40+ Treg (0.52) and OX40+Teff (r = 0.85) subsets. These associations remained strong when counts were normalized to total cells. Increase in prevalence of CD4+ (p = 0.019), total OX40+ (p = 0.046), and OX40+ Treg (p = 0.022) correlated with improved overall survival. Higher prevalence of OX40+ Teff cells also showed a trend of improved overall survival, but did not reach statistical significance.

## Conclusions

We have developed a multiplex immunofluorescence test to evaluate expression of OX40 in CD4+ T cell subsets and utilized it to determine associations between OX40+ cell subsets and clinical outcome. Our results show that higher number of total OX40+ cells and OX40+ Treg cells is associated with improved prognosis in CRC. Similar analyses in other indications are planned. Moreover, incorporating this test in clinical trials may identify patients that are likely to respond to therapeutics targeting OX40.

